# On the Design and Validation of Assessing Tools for Measuring the Impact of Programs Promoting STEM Vocations

**DOI:** 10.3389/fpsyg.2022.937058

**Published:** 2022-06-27

**Authors:** María Pilar Herce-Palomares, Carmen Botella-Mascarell, Esther de Ves, Emilia López-Iñesta, Anabel Forte, Xaro Benavent, Silvia Rueda

**Affiliations:** ^1^International PhD School (EIDUNED), National Distance Education University (UNED), Madrid, Spain; ^2^Department of Computer Science, Universitat de València, Burjassot, Spain; ^3^Department of Didactics of Mathematics, Universitat de València, València, Spain; ^4^Department of Statistics and Operational Research, Universitat de València, Burjassot, Spain

**Keywords:** diversity in STEM, gender stereotypes, informal education, self-efficacy, questionnaire validation, mixed methods

## Abstract

This paper presents the design and validation process of a set of instruments to evaluate the impact of an informal learning initiative to promote Science, Technology, Engineering, and Mathematics (STEM) vocations in students, their families (parents), and teachers. The proposed set of instruments, beyond assessing the satisfaction of the public involved, allow collecting data to evaluate the impact in terms of changes in the consideration of the role of women in STEM areas and STEM vocations. The procedure followed to develop the set of instruments consisted of two phases. In the first phase, a preliminary version (v1) of the questionnaires was designed based on the objectives of the Girls4STEM initiative, an inclusive project promoting STEM vocations between 6 and 18 years old boys and girls. Five specific questionnaires were designed, one for the families (post activity), two for the students (pre and post activity) and two for the teachers (pre and post avitivity). A refined version (v2) of each questionnaire was obtained with evidence of content validity after undergoing an expert judgment process. The second phase was the refinement of the (v2) instruments, to ascertain the evidence of reliability and validity so that a final version (v3) was derived. In the paper, a high-quality set of good practices focused on promoting diversity and gender equality in the STEM sector are presented from a Higher Education Institution perspective, the University of Valencia. The main contribution of this work is the achievement of a set of instruments, rigorously designed for the evaluation of the implementation and effectiveness of a STEM promoting program, with sufficient validity evidence. Moreover, the proposed instruments can be a reference for the evaluation of other projects aimed at diversifying the STEM sector.

## 1. Introduction

In recent years, multiple initiatives have emerged, from public and private institutions, to promote interest in disciplines related to Science, Technology, Engineering and Mathematics (STEM), especially among girls from an early age. These initiatives play a fundamental role in showing the relationship that exists between careers and professions in STEM areas and the generation of benefits in society. In addition, they serve to increase the visibility of proximity STEM female referents (UNESCO, 2017), helping to eliminate gender stereotypes (Sáinz et al., 2019).

The School of Engineering of the University of Valencia (ETSE-UV), in Spain, launched in 2011 a pilot program focused on increasing and retaining the number of Information and Communication Technology (ICT) female students in the institution (Botella et al., 2019). The results showed an increase in the proportion of female students in highly male-dominated ICT-related disciplines with a lower proportion of women in general (López-Iñesta et al., 2020). However, it was also observed that a degree such as Chemical Engineering, traditionally with a higher presence of women, showed a constant decrease in female enrollment. This suggested that a continuous effort was needed from educational institutions, public entities, professionals, and families to break the gender diversity gap in STEM (Sáinz and Müller, 2018; López-Iñesta et al., 2020).

The problem of the gender diversity gap in STEM disciplines, and specially in the ICT field, has been considered and analyzed from different perspectives (see Bian et al., 2017; Diekman et al., 2017, 2019; Sáinz and Müller, 2018; Botella et al., 2019; Sáinz et al., 2019; Benavent et al., 2020; López-Iñesta et al., 2020; Ayuso et al., 2021; Gladstone and Cimpian, 2021; Guenaga et al., 2022 and references therein). From these works, aspects such as the influence of gender stereotypes, the effectiveness of using role models, the concept of self-efficacy in STEM or understanding the impact of communal goal processes arise as fundamental factors to be covered by initiatives or programs focusing on pre-university students and aiming at diversifying STEM. There is a second pool of factors related to STEM working environments (i.e., perception of male-dominated environments, lack of work-life balance) which cannot be directly impacted by these type of initiatives. Instead, a large agreement between different social and economical actors should be sought.

In 2019, the Girls4STEM initiative was launched in the ETSE-UV as an evolution of the pilot program. The main feature of the project is that the target audience comprises pre-university students from 6 to 18 years old, as well as their families and teachers (Benavent et al., 2020). It is a project for both boys and girls, with an emphasis on girls, which is framed in the Sustainable Development Goals (SDGs) of United Nations and it is also aligned with the III Equality Plan of the University of Valencia (López-Iñesta et al., 2020). The specific objectives of the Girls4STEM initiative are: i) To awake curiosity about STEM disciplines from an early age; ii) To encourage the participation of students, teachers, families, and companies as a fundamental part of the project; iii) To give visibility to women developing their professional work in STEM areas and show their research, developments and progress; and iv) To increase the number of students in STEM studies through outreach activities such as seminars, workshops or interviews with leading women in STEM. The initiative is arranged around two main activities, Girls4STEM *family*, focused on pre-university students, their families and teachers, and Girls4STEM *Professional*, targeting a general audience. Note that a full description of the initiative can be found in Benavent et al. (2020). The initiative builds upon a large database of volunteer female STEM professionals, which are the ones interacting with the students and teachers via the *family* action or with the general audience via the *professional* action. The female STEM professionals act then as proximity role models, mitigating the impact of gender stereotypes, while the database helps increasing the visibility of their contributions to the society, reinforcing the link with communal goal objectives (Botella-Mascarell et al., 2021). In the *family* action, students gather with the STEM experts and they create 3 min videos about them which are later uploaded into the Girls4STEM YouTube channel. A contest is then arranged between the participating schools, where the Girls4STEM initiative selects the videos which best reflect the aims of the project.

The Girls4STEM initiative has been consolidated in two editions, being the edition 2021–2022 currently on-going. At this point, it is essential to have instruments with sufficient evidence of validity to evaluate with scientific rigor the impact of the initiative, as indicated by Tena Gallego and Couso (2019), beyond the satisfaction of the public involved. With this aim, this paper presents the design and validation process followed to obtain a set of instruments to evaluate the impact of the Girls4STEM initiative in the *family* action. To this end, the role of formal and informal learning contexts in STEM education is reviewed next, and the focus is then placed in informal education initiatives.

### 1.1. State of the Art

STEM education takes place in both formal and informal contexts and both need to be connected to promote students' STEM skills. Interestingly, informal education can overcome many of the shortcomings of formal education (Herce Palomares et al., 2022). Activities promoted by different initiatives or entities such as universities, museums, science fairs or contests are examples of informal education scenarios in which students, teachers, families or citizen participation is promoted (López-Iñesta et al., 2022). The audience and researchers/professionals in different fields can establish a useful bidirectional communication for fostering interest in STEM areas. From this point of view, the Girls4STEM initiative can be classified as an informal education/learning action organized by a Higher Education Institution. Girls4STEM builds bridges with formal education, involving both teachers and students' families from a systemic, integral and holistic educational vision. Although the word “informal” suggests insufficient correctness, it is actually highlighting the features of the learning environment. As pointed out in Allen and Peterman (2019), informal learning might contribute to achieve high levels of area-specific expertise for motivated student's. In addition, research suggests that educational experiences to promote STEM expertise in informal education play a decisive role (Herce Palomares and Román González, 2021) and, they also contribute to challenge common ideas and beliefs linked to STEM fields in formal education, as well as others related to scientific education (Benavent et al., 2020). In informal education learning, evaluation is one of the key components. Whilst helping to identify if aims and objectives have been met, it can also assist with planning, provide evidence of impact, and critically reflect for future engagement activities. Therefore, evaluation is a process that should run from the start of a project and continue after it has finished (Robinson and Murray, 2019).

Evaluating the impact in informal learning contexts poses a set of particular challenges (Habig, 2020). Firstly, evaluations should preserve the informal nature of science experiences, while defining appropriate evaluation metrics, using a common language, goals, and theories (National Research Council, 2009). Coupling these challenges with constraints on time, money, and operational capacity, the difficulty of obtaining meaningful, reliable and feasible evaluations becomes clear. The evaluation should then tackle these challenges to provide useful evidence-based information (Fu et al., 2016). Secondly, formal learning experiences are primarily intended to impart scientific knowledge and skills. However, informal learning experiences are intended to arouse curiosity, interest and encourage intrinsic motivation as “stepping stones” for STEM learning. This increases the difficulty of the evaluation process, since constructs such as interest, motivation and curiosity are more difficult to define, operationalize and measure (National Research Council, 2009). In this sense, evaluating the impact of educational interventions in informal STEM education requires the design of instruments that address the project objectives.

Three future directions for the measurement of the outcomes of informal STEM education actions are suggested in Grack Nelson et al. (2019). First, the measurement capacity should be enhanced. Currently, there is a small number of online repositories, covering also a limited range of activities and audience. Second, stronger collaborative networks should be established. These type of networks would allow to achieve shared measures combining different expertise (measurement experts, educational researchers, STEM experts). Finally, it is mandatory to increase the accessibility of shared measures. There are barriers related to intellectual property rights or instruments not accessible due to journal publishing options.

Another challenge related to the evaluation of the impact in informal STEM education is the broad range of projects and the large variety of methods used to conduct the evaluation. The most common form of evaluation is the user survey (Robinson and Murray, 2019). When designed well and interpreted appropriately, self-report surveys can be used to gather useful data from large samples at relatively low-cost (Wolf et al., 2021). Note that informal education initiatives are usually constrained by low budgets and hence, sustainable implementations should be sought. Therefore, in this work, the user survey technique via questionnaires is proposed to evaluate the impact of the Girls4STEM initiative in the *family* action, by designing and validating a set of questionnaires targeting pre-university students, their families and teachers.

**Figure 1:**
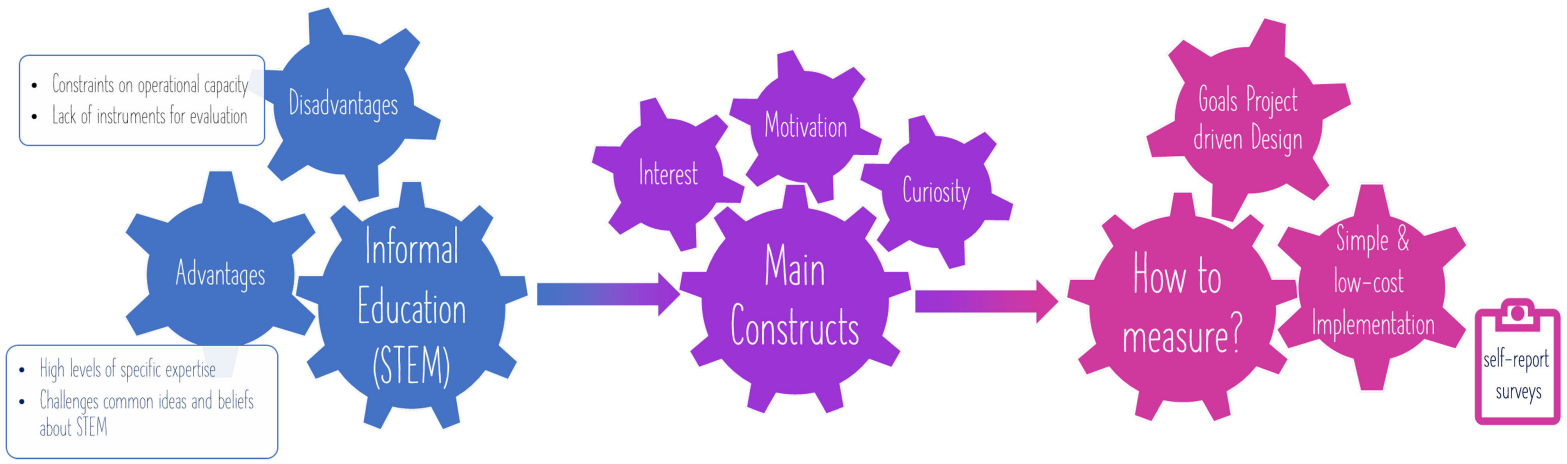
Advantages and disadvantages faced in STEM informal education, main constructs to measure and some hints about the evaluation.

With the increasing development and use of shared measures across the STEM education field, it comes the need for evaluators to better understand and assess instrument's technical qualities, in particular reliability and validity (Grack Nelson et al., 2019). On the one hand, the design of the evaluation instruments must be based on the objectives of the project. However, the questionnaires must undergo a validation process. Content validity evidence relates to how well the construct of interest is represented in the content of an instrument (Haynes et al., 1995; AERA, 2014). Such evidence can be collected by reviewing the literature and gathering feedback from experts related to the construct being measured. Experts review how the construct was defined, identify what is missing from the definition, and help to ensure that the essence of the items or tasks in the measure adequately cover the content area. On the other hand, evidence of the reliability of the questionnaires, after being administered to a pilot sample, is needed. Cronbach's alpha is commonly used to examine the internal consistency or reliability of summated rating scales (Cronbach, 1951; Cronbach and Shavelson, 2004; AERA, 2014), although there is an on-going discussion regarding its limitations (Trizano-Hermosilla and Alvarado, 2016; Xiao and Hau, 2022). Internal consistency describes the extent to which all the items in a test measure the same concept (or construct) and hence it is connected to the inter-relatedness of the items within the test. In addition to obtaining the reliability of the scale items, it is necessary to evaluate how open-response items work in the pilot sample. In this way, it is possible to check whether the answers given in the questionnaires have the same meaning for the target audiences as for the researchers interpreting the data (Wolf et al., 2021). Figure 1 summarizes the main advantages and challenges faced by STEM informal learning contexts, as well as the main constructs to measure and some hints about the instruments design.

### 1.2. The Present Study

This study tackles good practices focused on promoting gender diversity in the STEM sector from a Higher Education Institution perspective. A high-quality example of a gender-based intervention study in informal STEM education is presented, with sufficient evidence of the validity of a set of rigorously designed instruments for the evaluation of the implementation and effectiveness of the project. In addition, these instruments can be a reference for the evaluation of other projects aimed at reducing the gender diversity gap in STEM areas. The process and the results presented in this paper contribute to the directions suggested by (Grack Nelson et al., 2019), since the measurement capacity is increased, the questionnaires are accessible to other researchers and hence, there is potential to build a collaborative network. The main objective of this work is then to design and obtain evidence of reliability and validity of a set of instruments designed to evaluate the impact of the Girls4STEM initiative. This objective can be broken down into a set of specific objectives:

1. To design a set of questionnaires to evaluate the impact of the Girls4STEM initiative (*family* action). Each questionnaire will be specific for a different audience group: pre-university students, their families and teachers.
2. To obtain evidence of content validity of the set of questionnaires.
3. To obtain evidence of reliability of the set of questionnaires after administration to a sample and to assess whether the answers in self-assessment questionnaires have the same meaning for the target audiences and the researchers who interpret the data.

As discussed in the introduction, the gender diversity gap in STEM has been already considered from different perspectives. In Spain, the percentage of enrolled female students in the different STEM disciplines is not uniform. For example, in 2020-2021, there is a percentage of enrolled female students of 59.9% in life-sciences. In the case of Engineering, the number of enrolled female students goes down to 26.1%, and to 14.2% in the case of Computer Science^1^. There are several initiatives or projects located in Spain that work toward diversifying the STEM sector (Botella et al., 2020). Most of them can be classified as informal education actions, and they also face the evaluation challenges discussed above. Note that some of these initiatives are nodes from international projects. Some representative examples in Spain are, first of all, the Inspira STEAM Program, which is a mentoring program for students between the ages of 10 and 12 years. Results of the program showed an impact on the students' attitudes toward technology, an increase in the number of female STEM referents the student's knew, and an improvement of the students' opinion regarding vocations and professions related to science and technology. Moreover, a larger impact was measured among girls (Guenaga et al., 2022). Secondly, the program by the Inspiring Girls Foundation focuses on pre-university 12–16 years old girls, which interact with female role models working in STEM fields. Reference (González-Pérez et al., 2020) shows a set of benefits on mathematics enjoyment, importance attached to math, expectations of success in math, and girls' aspirations in STEM, and a negative effect on gender stereotypes, among others. Thirdly, the project Science and Technology as Feminine aims at students in the 1st to 3rd years of compulsory secondary education (therefore aged 11–14 years). Results in Santos et al. (2021) show that it should be possible to reduce the gender gap in the future career choices of young students, through the design of a set of activities addressed to individual students, the students' families and peers, schools and society at large, aimed at changing the habits, which for many years have steered women away from STEM. Despite the relevance and impact of the above STEM education initiatives, there is a lack of instruments with evidence of reliability and validity to assess the impact of the projects themselves, since they either make use of questionnaires to measure specific dimensions (i.e., gender stereotypes (Colás Bravo and Villaciervos Moreno, 2007), mathematical self-efficacy (Schwarzer and Baessler, 1996) and attitudes toward technology (Kier et al., 2014)) or questionnaires without a sufficient design and validation process. To the best of our knowledge, this paper contributes to the state of the art of informal STEM education by providing the description of the process and evidences of reliability and validity of a set of instruments that were designed to specifically assess Girls4STEM's objectives.

The paper is organized as follows. Section 2 presents the two phases followed for the design and validation of the proposed set of instruments. Details about the samples used in each one of the phases are given and the data analysis approach followed is explained. The section finishes providing the results obtained in terms of content validity and reliability for the set of instruments. Finally, section 3 discusses the main findings of this research.

## 2. Materials and Methods

The present work uses a Mixed Methods Research (MMR) approach whereby both qualitative and quantitative data are collected and analyzed in the same study. MMR is often used in social and behavioral studies, such as education or health, to strengthen the reliability of qualitative data, allowing to put quantitative results in a context and enriching the findings and conclusions (Creswell and Clark, 2003; Onwuegbuzie and Johnson, 2006; Anguera et al., 2012; Fàbregues et al., 2019). In the specific context of this work, using mixed methods can both increase the validity and reliability of the data collected with the designed instruments and improve the evaluation procedure to measure the impact of the initiative (Shekhar et al., 2019; Griffiths et al., 2021; Hargraves et al., 2021. In this sense, the aim of the study is to design and validate a set of different instruments for measuring the impact on students, parents and teachers of a program promoting STEM vocations that can be used on a large scale by other researchers.

**Figure 2:**
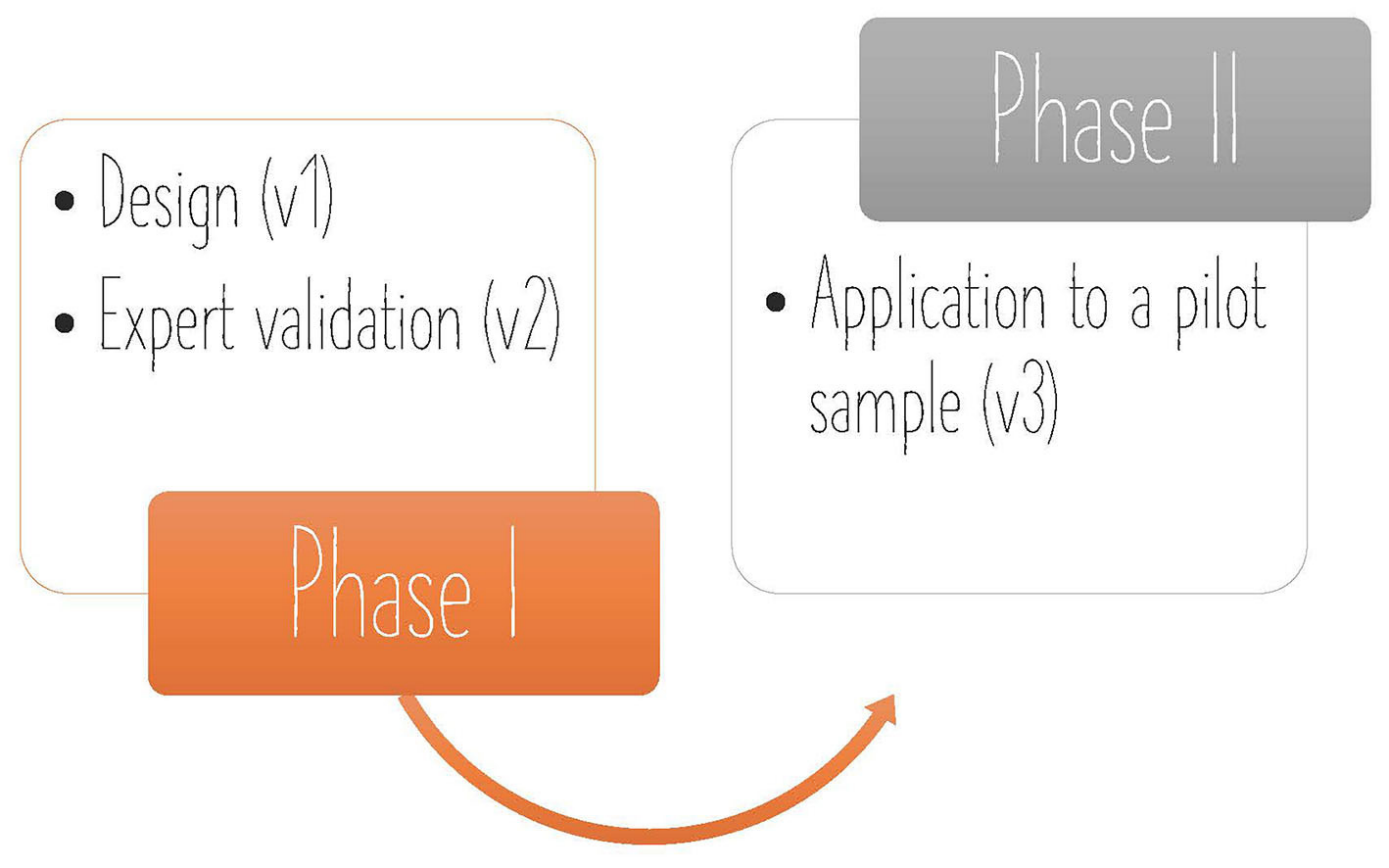
Phase I and phase II stages, and questionnaire versions obtained in each one of them.

The procedure consisted of two phases. First, in phase I, a preliminary version of the questionnaires was designed by the leading researcher based on the objectives of the Girls4STEM initiative, obtaining a first version (v1) of each one. Afterwards, 6 experts participating in the project and with experience in instrument construction and validation, modified and/or polished the items of the different questionnaires through an expert judgment process to obtain evidence of content validity, deriving the version (v2). In the second phase, phase II, the version (v2) instruments were distributed to a pilot-sample. Evidence of reliability was gathered and a final refinement process was carried out. Finally, the final version (v3) was obtained. All the questionnaires collected socio-demographic information and some indicators with a response format with open-ended, multiple choice answers and Likert scale options (1 to 5). Figure 2 summarizes the steps followed during the process of design and validation of the instruments.

### 2.1. Instrument: Design and Validation Process

In this subsection, the two-phase process for obtaining the instruments is detailed. Note that there are a total of five questionnaires targeting different groups: parents (post-activity), students-pre (prior to activity), students-post (post-activity), teachers-pre (prior to activity) and teachers-post (post-activity). The first instrument is a questionnaire for families, administered once the participation in the project is finished. It includes indicators on the overall impact of the initiative and on the individual (family member). An indicator is also provided on the possible improvement of the project and the promotion of STEM within the family. Secondly, there are two questionnaires for students that are applied before and after participating in the project. The pre questionnaire collects indicators on STEM interests, their perception of STEM competence and performance in STEM subjects. The post collects indicators on the degree of participation, the impact and possible improvement of the project. The teachers' questionnaires are also arranged in pre and post. The pre includes indicators on motivation and expectations of the project. The post questionnaire asks about their participation degree, the project impact, and suggestions for improvement.

**Phase I. Design and evidence of content validity using the expert judgment method**. The first phase consisted of two parts. Firstly, an initial version (v1) of the questionnaires was designed by the leading researcher and secondly, evidence of content validity using the expert judgment method was obtained, after which a new version (v2) of each of the five questionnaires was available.
  The five questionnaires in their initial version (v1) were designed using as a reference the objectives of the Girls4STEM initiative. A set of items was generated to collect inputs from the subjects participating in the *family* action (families/parents, students and teachers), and the dimensions to be measured according to the objectives were specified. An *ad hoc* questionnaire for each of the five questionnaires was then prepared, which was distributed to the committee of experts for undergoing the expert judgment process. These *ad hoc* questionnaires asked about the pertinence/representativeness (whether the items are representative of the dimensions they are intended to measure), relevance (whether the items contribute with important information to the measurement of the dimension) and formulation (whether the items are understood, unambiguous and clear), all on a Likert scale from 1 (not at all in agreement) to 6 (totally in agreement).
  In addition, after each set of items, suggestions were requested in open-ended questions when not in complete agreement and an open-ended question was provided at the end of each questionnaire, for any relevant considerations on the design of the instrument. The five *ad hoc* questionnaires were distributed to the committee of experts online, and they were sent to them as well in advance, so that the five questionnaires could be accessed before making their judgments.
**Phase II. Distribution of the instruments to a pilot sample**. In the second phase, the five instruments in version (v2) were administered through non-probabilistic purposive sampling to a pilot sample of families (parents), students and teachers participating in Girls4STEM in the 2020–2021 academic year. Before the start of the project and the distribution of each questionnaire, informed consent was requested and the current legislation on data protection was complied with, while maintaining the confidentiality of the data. A double analysis (quantitative and qualitative) was performed with the results. First, with the quantitative information, the reliability as internal consistency was calculated from the two-factor model based on the average correlation between the items, using the SPSS v27 program (George and Mallery, 2010), and studying the items on a Likert scale. Secondly, the open-ended questions were analyzed by the group of researchers by means of a content analysis to determine how the questionnaire worked in the population and to be refined if necessary.

### 2.2. Sample

In this subsection, a description of the sample of each one of the phases is provided.

**Phase I**. Six female researchers made up the committee of experts. This is a non-probabilistic purposive sample, all of them being women. The selection meets the criteria proposed by Skjong and Wentworth (2000) for purposive sampling: experience in making judgments and decisions based on evidence or expertise, reputation in the community, availability and motivation to participate, impartiality and inherent qualities such as trustworthiness and adaptability.
**Phase II**. A total of 8 schools, all of them located in the Valencian Community, participated in the Girls4STEM initiative during the 2020–2021 academic year. From these schools, 6 were public and 2 were charter schools. Regarding their geographical origin, 2 of them were located in small cities (*population* < 30, 000), 3 in medium-sized cities (*population* < 100, 000), while 3 were located in large cities (*population* > 100, 000). This brings the total group of students participating to 298, distributed between 84 in small cities, 109 in medium-sized cities and 105 in large cities.
  The final sample used for this study, eliminating those students who did not fill in the pre or post questionnaires, was 268 students, 18 teachers (16 female and 2 male teachers) and 113 family members (88 female and 25 male). Therefore, the sample was constructed by non-probability purposive sampling.
  Table 1 shows the distribution of participating students according to gender, with a higher percentage of female students (62%), and educational level, defining the following levels: primary, secondary with 2 subgroups by age, and professional studies.
  Regarding the education level, the table shows that the largest group was secondary education with students between 12 and 16 years old, accounting for 78% of the total sample. The educational level with the lowest representation in our sample corresponded to secondary education, aged 17–18 (0.03%).

**Table 1:** Number of students who completed the pre and post questionnaires by gender and educational level.

	**Gender**			
**Educational level**	**Male**	**Female**	**Undeclared**	**Total**
**Primary**	16	15	1	32
Secondary (12–16 years old)	74	135	1	210
Secondary (17–18 years old)	5	4		9
Professional studies	5	12		17
Total	100	166	2	268

### 2.3. Data Analysis

Data have been processed according to the specific objectives of the research and the established phases. A description of the process followed in each phase is included in this subsection.

**Phase I**. The SPSS version 27 software was used to calculate the evidence of content validity. Firstly, the mean of the items of each questionnaire in the three dimensions under evaluation (representativeness, relevance, and formulation) was obtained. Given that the Likert scale consisted of 6 points, the criterion for refining an item was that a mean less than 5 were obtained (a value of 5 suggested agreement and 6 suggested total agreement). Secondly, the internal consistency of the judgments issued was calculated by obtaining Cronbach's alpha as intraclass correlation coefficients, according to the bidirectional random model of consistency suggested by Gwet (2014). Finally, the Content Validity Ratio (CVR) of each item was calculated by applying the model of Lawshe (1975) modified by Tristán-López (2008):
  CVR=ne/N,
  where *ne* is the number of experts who gave a favorable judgment (5 or 6 in representativeness) and *N* is the total number of experts who responded to the *ad hoc* questionnaire. The CVR provides evidence of content validity for each indicator. From this model, items are considered essential when scores of 5 and 6 are obtained on the Likert representativeness scale. Any item with a score lower than 0.58 should be deleted (Tristán-López, 2008). The *ad hoc* questionnaires also offered open-ended questions to complete the assessments. In the event that an item needed to be refined, it was modified according to the suggestions of the experts.
**Phase II**. The data collected after the administration of the version (v2) instruments to a pilot sample of subjects (parents, teachers and students) was analyzed. With the quantitative information (Likert scale questions), Cronbach's alpha reliability coefficient was calculated. With the qualitative information, a content analysis was conducted, in order to assess the performance of the instruments in the sample and to refine them if necessary. Groenvold et al. (1997) suggests that, although rarely investigated, it is necessary to check whether the answers in self-assessment questionnaires have the same meaning for the target audiences as for the researchers who interpret and report the data.

## 3. Results

This section presents the results of the design and debugging process of the five questionnaires. Results of phase I provide evidence of content validity after the design process, for each of the five questionnaires. Results of the phase II include evidence of reliability of the scale items and an analysis of the performance of the qualitative items.

### 3.1. Phase I

First, the results related to the specific objectives 1 and 2 of the paper are presented. Table 2 summarizes the questionnaires in version (v1) including the dimensions, items and scale used in each one. The questionnaires collected the information that was considered appropriate for the measurement of the initiative's objectives, although for the objective of *increasing the number of students in STEM studies*, an indirect measurement of the results was proposed, by assessing interest at the time of the evaluation. As it can be seen in the table, the evaluation was not limited to measuring participant satisfaction. For each set of participants, the measurement of those aspects that were considered critical was proposed. In addition, indicators were included on issues relevant to achieving the aims of Girls4STEM, which are intended to be analyzed in further research, such as family involvement in promoting STEM interests, factors that contribute to student involvement in STEM studies, such as achievement or interest (UNESCO, 2017), or the role of teachers in promoting STEM vocations. Note that the questionnaires collected information on socio-demographic data, which is out of the scope of this study.

**Table 2:** Design of the questionnaires (v1).

**Questionnaire**	**Dimensions (item number)**	**Scale**
Parents	Overall impact (1–3)	2 multiple choice
		1 dichotomous (with open-ended question)
	Impact on parents (4–7)	4 Likert (1–5 points)
	Satisfaction and project improvement (8–10)	1 Likert (1–5 points)
		2 open-ended questions
Students-pre	STEM interests (1–2)	1 dichotomous (with open-ended question)
		1 Likert (1–5 points)
	Achievement in STEM subjects (3)	1 open-ended question
Students-post	Degree of participation (1–2)	2 open-ended questions
	Impact on students (3–6)	4 Likert (1–5 points)
	Satisfaction and project improvement (7–9)	1 Likert (1–5 points)
		2 open-ended questions
Teachers-pre	Motivation toward the project (1–2)	2 open-ended questions
	Expectations (students) (3–5)	3 open-ended questions
	Expectations (teachers) (6)	1 open-ended questions
Teachers-post	Degree of participation (1–2)	2 open-ended questions
	Impact on students (3–5)	3 open-ended questions
	Impact on teachers (6–13)	1 open-ended question
		1 multiple choice
		6 Likert (1–5 points)
	Satisfaction and project improvement (14–15)	1 Likert (1–5 points)
		2 open-ended questions

*Dimensions and items, and scale of each one are included in the second and third row, respectively*.

After the design of the questionnaires in their initial version (v1), the questionnaires were subjected to expert judgment to reach evidence of content validity and to refine the questionnaires, if necessary. *Ad hoc* questionnaires were distributed for expert judgment, and the obtained results for the inter-rater reliability (Cronbach's alpha) are summarized in Table 3. In the following, the evidence of content validity is discussed for each questionnaire, both considering the mean of the items of each questionnaire and the internal consistency of the judgments.

**Evidence of content validity of the parents questionnaire**. The questionnaire for parents (v1) consisted of a total of 10 items (see Table 2). The results in terms of the mean of the items after the expert judgment are shown in Table 4. In the dimension of representativeness, the mean of all the items ranged between 5.67 and 6, so none of them had to be modified, according to the criterion defined beforehand. Cronnbach's alpha coefficient in Table 3 suggested sufficient consistency with a value of 0.262. Finally, the CVRs for all the items were 1, which leaded to the conclusion that the questionnaire had sufficient evidence of content validity in the representativeness dimension, i.e., the items were representative of the dimensions they were intended to measure. In the relevance dimension, the results were similar to the ones in the representativeness dimension, with means between 5.67 and 6 (Table 4) and a Cronbach's alpha as intraclass correlation of 0.4 (Table 3). The formulation dimension pointed in another direction. Both item 2 and 4 showed values below 5, so both needed to be reformulated. In spite of this, this dimension presented a high consistency, since Cronbach's alpha value was 0.895. In order to proceed with the refinement, the open-ended questions were analyzed qualitatively. In item 2, two experts suggested introducing “in his/her family” and in item 5, replacing “the role” with “participation.” The suggestions were accepted and both items were reformulated.
**Evidence of content validity of the students-pre questionnaire**. The initial student questionnaire (v1) consisted of three items (see Table 2), although the first item offered a dichotomous response which, if affirmative, required an explanation in an open-ended question. Table 5 shows the results of the mean of the items after the expert judgment for each dimension. In the representativeness dimension, the mean of the items ranged between the values 5.33 and 5.67 (no rephrasing of any of the items necessary). These results were consistent with a Cronbach's alpha of 0.8 (Table 3). In addition, none of the items needed to be deleted in terms of the CVR criterion, since all of them reached the maximum value (CVR = 1, except item 2 with CVR = 0.83, which also exceeded 0.58). In the dimension of relevance, Cronbach's alpha (Table 3) again suggests consistency in the judgments (Cronbach's alpha = 0.6). The means were higher than in the previous dimension, with values between 5.67 and 6. However, as in the questionnaire for families, the dimension of formulation showed a very high consistency (Cronbach's alpha = 0.944 in this case), but the means indicated the need to reformulate item 2 (mean = 4.17) and 3 (mean = 4.5). Therefore, the open-ended questions of the expert judgment that explained this result were studied. Given that in the Spanish educational system the subjects in the primary and secondary education stages related to STEM contents are different, the experts proposed to specify the term “STEM” in the curricular subjects of both indicators and to not limit the answers to primary education subjects. For version (v2) of this questionnaire, STEM interests (item 2) and school performance (item 3) were defined on the basis of these subjects. Finally, in the open-ended questions at the end of the *ad hoc* questionnaire, it was suggested to incorporate a new dimension, the self-efficacy (perceived achievement), as the experts judges considered it to be a relevant indicator in STEM education. A new indicator was added as requested by the experts.
**Evidence of content validity of the students-post questionnaire**. The final student questionnaire (v1) consisted of 9 items (see Table 2). Table 6 shows the results of the mean of the items after the expert judgment en each dimension. In the dimension of representativeness, item 1 and 2 were below the criterion (at least 5). In addition, the results showed a high consistency (Cronbach's alpha = 0.987) and the CVR warned about a low-content validity of the first two items, since CVR = 0.33 and CVR = 0.17 for item 1 and 2, respectively. This indicated that both items should be deleted. The experts' feedback on the open-ended questions was reviewed. In item 1, they considered that it was not a decision for the students to take, so the item was not appropriate. For item 2, both in this dimension and in relevance, they suggested incorporating the measurement of the degree of participation with new indicators such as justifying participation in the specific project, and quantitatively specifying the degree of participation in number of hours. Items 1 and 2 were eliminated and two new items were created to evaluate the degree of participation. The information obtained in the results for relevance was similar to the representativeness dimension, with the first two items of the degree of participation being the ones that need to be modified. The means of the items 1 and 2 were again below the criterion. Cronbach's alpha reached a high value (Cronbach's alpha = 0.981) and the open-ended questions raised the point found in the dimension of representativeness. Both items 1 and 2 were reformulated. The formulation dimension showed much more satisfactory results, as all the means were above the criterion and Cronbach's alpha = 0.273, so the consistency was sufficient. No item was subject to change after the results in the formulation. However, in the open-ended question of the final part of the *ad hoc* questionnaire, two experts suggested changing the order of presentation of items 4 and 5. They argued that item 5 was related to the interests raised in item 3, although in this case in relation to the professions. The suggested change in the presentation format was included.
**Evidence of content validity of the teachers-pre questionnaire**. The initial teacher questionnaire (v1) consisted of 6 open-ended questions items (see Table 2). Table 7 shows the results of the mean of the items after the expert judgment for each dimension. In the representativeness dimension, only item 2 was below the criterion and needed refinement. The inter-rater reliability was sufficient (Cronbach's alpha = 0.935), but item 2 showed a CVR = 0.33, which indicated that the item should be removed from the questionnaire. Item 4 showed a CVR = 0.66, but it was kept in the questionnaire since it exceed the criterion of 0.58 (Tristán-López, 2008). In the dimension of relevance, item 2 was also below the criterion. The judgments were consistent, since Cronbach's alpha = 0.946. Finally, the formulation dimension did not require modification, since the means were above the criterion and Cronbach's alpha = 0.359. In summary, item 2 was eliminated, and version (v2) was composed of 5 items.
**Evidence of content validity of the teachers-post questionnaire**. The final teacher questionnaire (v1) consisted of 16 items (see Table 2). Table 8 shows the results of the mean of the items after the expert judgment for each dimension. In the dimension of representativeness, item 2 was below the criterion. The results were consistent with Cronbach's alpha = 0.69. The CVR of all the items was 1, except for item 2 where CVR = 0.66. Since this value exceeded the criterion of 0.58, the item did not need to be removed. The results in the dimension of relevance were larger, but item 2 was below the criterion. The judgments were consistent with a Cronbach's alpha = 0.92. In the formulation dimension, the results were similar to the other dimensions, with a Cronbach's alpha = 0.942 and the mean of item 2 below the criterion. The judges open-ended responses were revised for item 2. The suggestion was to divide item 2 and quantify it. Hence, the item 2 (“How much time have you spent on it and how much time have your students spent on it?”) was divided in two new items: “Indicate the number of hours you have spent” and “Number of videos in which you have participated.” After the modification, version (v2) was composed of 17 items.

**Table 3:** Inter-rater reliability (Cronbach's alpha).

**Questionnaire**	**Dimension**	**Cronbach's alpha**
Parents	Representativeness	0.262
	Relevance	0.406
	Formulation	0.895
Students-pre	Representativeness	0.8
	Relevance	0.6
	Formulation	0.944
Students-post	Representativeness	0.987
	Relevance	0.981
	Formulation	0.273
Teachers-pre	Representativeness	0.935
	Relevance	0.946
	Formulation	0.359
Teachers-post	Representativeness	0.69
	Relevance	0.92
	Formulation	0.942

**Table 4:** Mean (parents).

**Mean**	**Item 1**	**Item 2**	**Item 3**	**Item 4**	**Item 5**	**Item 6**	**Item 7**	**Item 8**	**Item 9**	**Item 10**
Representative	5.83	6	5.83	6	5.67	6	6	5.67	6	6
Relevance	5.83	6	5.67	6	5.67	6	6	6	6	6
Formulation	6	4	5.83	5.83	4.17	6	6	6	6	6

**Table 5:** Mean (students-pre).

**Mean**	**Item 1**	**Item 2**	**Item 3**
Representative	5.67	5.33	5.33
Relevance	5.67	5.67	6
Formulation	6	4.17	4.50

**Table 6:** Mean (students-post).

**Mean**	**Item 1**	**Item 2**	**Item 3**	**Item 4**	**Item 5**	**Item 6**	**Item 7**	**Item 8**	**Item 9**
Representative	3.17	3	6	5.83	5.67	6	6	6	6
Relevance	2.50	2.83	5.83	6	5.50	6	5.67	5.83	5.83
Formulation	5.83	6	5.67	5.83	6	6	6	6	6

**Table 7:** Mean (teachers-pre).

**Mean**	**Item 1**	**Item 2**	**Item 3**	**Item 4**	**Item 5**	**Item 6**
Representative	5.67	3.17	5.67	5	5.67	6
Relevance	5.67	3	5.67	5	5.5	6
Formulation	5.83	5.50	6	6	6	5.83

**Table 8:** Mean (teachers-post).

**Media**	**Item 1**	**Item 2**	**Item 3**	**Item 4**	**Item 5**	**Item 6**	**Item 7**	**Item 8**	**Item 9**	**Item 10**	**Item 11**	**Item 12**	**Item 13**	**Item 14**	**Item 15**
Representative	5.67	4.67	5.67	5.5	6	6	5.67	6	5.83	5.83	5.67	6	5.83	6	5.5
Relevance	5.67	3.83	5.67	5.67	6	6	5.83	6	6	5.83	5.83	6	5	6	5.5
Formulation	6	3.7	5.83	5.83	5	6	6	6	5.17	5.83	6	6	5.83	6	6

Once phase I was completed, all five questionnaires were available in version (v2), with sufficient evidence of content validity in all of them.

### 3.2. Phase II

In order to collect data for phase II of this study, the pre-questionnaires were administered to students and teachers before interacting with the STEM experts, so gender and professional career aspects have not yet been discussed. The post-questionnaires for students, teachers and families (parents) were administered after each school submitted the STEM expert biography video to the initiative. All the questionnaires were delivered using the Microsoft forms platform. In the following, results related to the specific objective 3 of the paper are analyzed both quantitatively and qualitatively.

#### 3.2.1. Evidence of Reliability

The aim was to ascertain the evidence of reliability and to refine the questionnaires if necessary. To this end, the results were analyzed quantitatively. Table 9 summarizes the dimensions, scale and analysis type of the different version (v2) questionnaires. The quantitative information was used to determine the evidence of reliability. To this end, reliability was calculated as internal consistency (using the SPSS v27 program), from the two-factor model based on the average correlation between the items that were formulated using a Likert scale. As it can be seen in Table 9, this analysis was feasible for all the questionnaires except for the teachers-pre case. Table 10 shows the results of evidence of reliability for each one of the questionnaires. The second column indicates the number of items that were evaluated (formulated using a Likert scale), the third column stands for the number of valid samples used out of the total number of responses collected from the pilot sample and the fourth column gives the value of the Cronbach's alpha coefficient. In the fifth column, the evaluated item number is provided, while column 6 shows the total correlation of the corrected item and finally, column 7 gives the Cronbach's alpha coefficient if the item is deleted. Note that item 11 of teachers-post questionnaire did not offer results after its calculation, since the answers of all the subjects presented the same value, in this case 5.

**Table 9:** Design of the questionnaires (v2).

**Questionnaire**	**Dimensions (item number)**	**Scale**	**Analysis type**
Parents	Overall impact (1–3)	2 multiple choice	Qualitative
		1 dichotomous (with open-ended question)	Qualitative
	Impact on parents (4–7)	4 Likert (1–5 points)	Quantitative
	Satisfaction and project improvement (8–10)	1 Likert (1–5 points)	Quantitative
		2 Open-ended questions	Qualitative
Students-pre	STEM interests (1–2)	1 dichotomous (with open-ended question)	Qualitative
		1 Likert (1–5 points)	Quantitative
	Self-efficacy: perceived achievement (3)	1 Likert (1–5 points)	Quantitative
	Achievement in STEM subjects (4)	1 open-ended question	Qualitative
Students-post	Degree of participation (1–2)	2 open-ended questions	Qualitative
	Impact on students (3–6)	4 Likert (1–5 points)	Quantitative
	Satisfaction and project improvement (7–9)	1 Likert (1–5 points)	Quantitative
		2 open-ended questions	Qualitative
Teachers-pre	Motivation toward the project (1)	1 open-ended question	Qualitative
	Expectations (students) (2–4)	3 open-ended questions	Qualitative
	Expectations (teachers) (5)	1 open-ended question	Qualitative
Teachers-post	Degree of participation (1–3)	2 open-ended questions	Qualitative
		1 multiple choice answer	Qualitative
	Impact on students (4–6)	3 open-ended questions	Qualitative
	Impact on teachers (7–14)	1 open-ended question	Qualitative
		1 multiple choice answer	Qualitative
		6 Likert (1–5 points)	Quantitative
	Satisfaction and project improvement (15–17)	1 Likert (1–5 points)	Quantitative
		2 open-ended questions	Qualitative

*The second column includes the dimensions and the item number in parentheses. The scale and the type of analysis are included in the third and fourth column, respectively*.

**Table 10:** Summary of the Cronbach's alpha results in phase II.

**Questionnaire**	**N of items**	**N valid / N samples**	**Cronbach's alpha**	**Items**	**Corrected item**	**Cronbach's alpha**
		**(cases)**		**(questionnaire)**	**(Total correlation)**	**if item deleted**
Parents	5	112 / 113	0.85	4	0.55	0.85
				5	0.73	0.80
				6	0.73	0.80
				7	0.67	0.81
				8	0.63	0.83
Students-pre	4	32 / 32	0.49	2A	0.42	0.25
(Primary)				2B	0.05	0.59
				3A	0.43	0.24
				3B	0.25	0.45
Students-pre	6	218 / 236	0.82	2A	0.56	0.79
(Secondary)				2B	0.52	0.80
				2C	0.66	0.77
				3A	0.55	0.79
				3B	0.56	0.79
				3C	0.61	0.78
Students-post	5	220 / 220	0.8	3	0.67	0.73
				4	0.65	0.74
				5	0.58	0.76
				6	0.36	0.82
				7	0.67	0.73
Teachers-post	6	14 / 14	0.65	9	0.33	0.63
				10	0.18	0.66
				11	-	-
				12	0.07	0.68
				13	0.71	0.46
				14	0.66	0.47
				15	0.49	0.61

George and Mallery (2010) suggest that, in order to evaluate the values of Cronbach's alpha coefficients, a value above 0.7 is considered acceptable. Loewenthal and Lewis (2001) warns that, in scales with less than 10 items, an internal consistency value of 0.6 can be considered acceptable. Results in Table 10 show that sufficient evidence of validity was achieved in all the questionnaires in the sample used, except for the students-pre questionnaire, with a Cronbach's Alpha = 0.49, which is a low value. The study of the corrected item-total correlation pointed out that item 2B presented a low linear correlation between this item and the total score of the scale. Moreover, Cronbach's alpha improved if this item was deleted. However, the item was kept, since it was actually the same question posed in 2A, but applied to the subject of natural sciences, instead of mathematics. In addition, it should be noted that having only 4 items in this questionnaire may have contributed to the low Cronbach's alpha coefficient.

#### 3.2.2. Analysis of Qualitative Information

The goal is to provide meaningful feedback about the respondents' thought processes when responding to survey items. Then, it is necessary to gather evidence that survey items and response options are well understood by respondents Wolf et al. (2021). From the qualitative data, the answers given by all the participants were analyzed in parallel by each researcher to determine how the questionnaires worked in a real sample and to refine items if necessary. Researchers assessed the following questions for the items that had not been answered on a Likert scale in each questionnaire:

q1. If the item was understood and corresponded to the measured dimension. In this way, it is possible to have evidence of face validity i.e., to recognize the pertinence of the evaluation system by analyzing the answers given. The researchers indicated yes or no. In case of a negative answer, the reasons were noted down.
q2. If there were responses that could suggest presenting the item in another format or with some change in its presentation, in order to improve it. If they considered it appropriate, they suggested the reasons.
q3. Observations, if they considered any comment necessary, when they had answered “no” in any of the previous items.

Table 11 synthesizes by questionnaires and items the proposals of the group of 6 researchers. The columns “Relevance of the evaluation system” and “Presentation format” indicate the number of yes respondents from the 6 researchers. The last column, “comments,” includes the observations when the researchers disagreed or any other comments they considered of interest.

**Table 11:** Qualitative analysis (v2).

**Questionnaire**	**Dimensions**	**Item**	**Relevance**	**Presentation**	**Comments**
				**format**	
Parents	Overall impact	1	6	6	No answer “nothing” or “other”
		2	6	6	No answer “other”
		3	6	6	
	Satisfaction and project improvement	9	6	6	
		10	6	6	
Students-pre	STEM interests	1	6	6	
	Achievement in STEM subjects	4	6	3	Modify to closed response (multiple choice)
Students-post	Degree of participation	1	6	6	
		2	6	3	Modify to closed response (multiple choice)
	Satisfaction and project improvement	8	6	6	
		9	6	6	
Teachers-pre	Motivation toward the project	1	6	6	
	Expectations (students)	2	6	6	
		3	6	6	
		4	6	6	
	Expectations (teachers)	5	6	6	
Teachers-post	Degree of participation	1	6	6	
		2	6	3	Modify to closed response (multiple choice)
		3	6	6	
	Impact on students	4	6	6	
		5	6	6	
		6	6	5	Add: “justify your answer”
					(some subjects indicate “positively” without explanation)
	Impact on teachers	7	6	6	
		8	6	6	
	Satisfaction and project improvement	16	6	6	
		17	6	6	

*Dimensions and number of items are included in the second and third column, respectively. The fourth and fifth columns collect the number of positive answers in each dimension, relevance and presentation format, respectively. Observations raised by the researchers are included in the last column*.

In general terms, it can be seen that all the responses to the items building the questionnaires met the objective for which they were designed, since all six researchers agreed that, after analyzing all the results, there was no response that did not meet the indicator. They also agreed that the presentation format was adequate in most of the items, but some needed to be revised. Fifty percent of the researchers proposed to modify the type of response in three items: i) in the initial questionnaire for students, item 4 (performance in STEM subjects); ii) in the final questionnaire for students, item 2 (degree of participation); and iii) in the final questionnaire for teachers, item 2 (degree of participation). In addition, other comments were raised in item 1 and 2 of the overall impact on parents, since some of the multiple-choice answers were not chosen, as indicated in the table. Following the parallel analysis, the researchers participated in a debriefing until a consensus was reached on the changes needed. The results and conclusions of the discussion were as follows:

- **Parents questionnaire**. One of the researchers suggested that some of the multiple-choice options were not selected by any subject. Although she considered that the presentation format was adequate, she offered this topic for discussion. Researchers agreed that since there was a possibility that some person may point out these options in another sample, the presentation format should be maintained.
- **Students-pre questionnaire**. Fifty percent of the researchers suggested modifying the presentation format in the achievement in STEM subjects (item 4). In the discussion it became clear that it was a numerical response and that the open response option caused some students to indicate values with decimals, others in intervals, others suggested not remembering their grade and even subjective sentences such as “very bad grade”. In the Spanish educational system, in secondary education, the optional nature of some subjects means that they are not prescriptive for all students. Therefore, in order to improve the coding and interpretation of the results, researchers agreed to present this item as a multiple-choice response with the following options: 0–3, 3.1–4.9, 5–5.9, 6–6.9, 7–8.9, 9–10, and *I do not take this course*.
- **Students-post and teachers-post questionnaires**. Item 2 measuring the degree of participation was discussed in both questionnaires. Fifty percent of the researchers suggested a closed response. Similar to the students-pre questionnaire discussion, a multiple-choice presentation format was decided, since it was seen that some answers provided intervals of hours of participation, or subjective sentences such as “many” or “the class hours.” To avoid difficulties in processing the information, the multiple options were specified as follows: 0–1 h, between 1 and 2 h, between 2 and 3 h, between 3 and 4 h, between 4 and 5 h, between 5 and 6 h, between 6 and 7 h, between 7 and 8 h, between 8 and 10 h, between 10 and 15 h and more than 15 h. These intervals were established based on the analysis of the answers given in the pilot sample. Finally, in the teachers-post questionnaire, a researcher suggested including “justify your answer” in item 6 on the impact on students, since she appreciated that some of the answers evaluated the project “positively” without providing arguments. The suggestion was accepted by the rest of the researchers, so the formulation of the question was modified.

## 4. Discussion

The research presented in this paper aims at contributing to the state of the art of informal STEM education by describing the process of how to obtain evidences of reliability and validity of a set of instruments. This set of instruments comprises five questionnaires for the evaluation of the impact of the *family* action from the Girls4STEM initiative, which includes all the participants: students, families (parents) and teachers. The initial specific objectives of this research have been fulfilled. Firstly, in phase I, the initial version (v1) of the questionnaires has been designed, considering the initiative's objectives and important dimensions to measure. The five questionnaires have been subjected to an expert judgment, to obtain evidence of validity of these instruments and to refine them if necessary. The results of all of them suggest high content validity through the calculation of the CVR, means and inter-rater reliability, which confirms the consistency of the results. Nevertheless, it has been necessary to delete some of the items, as well as to reformulate others. Specifically, the following changes have been necessary in the debugging process:

- Parents questionnaire: reformulation of items 2 and 5, given their means in the formulation dimension.
- Students-pre: reformulation of items 2 and 3, given their means in the formulation dimension. In addition, a new item on perceived achievement in STEM subjects has been added.
- Students-post: deletion of items 1 and 2, due to their CVRs values and their low means in representativeness and relevance. Two new items have been constructed from open-ended questions to determine the degree of participation (given that former items 1 and 2 were dealing with this metric). The order of items 4 and 5 has been changed, following the proposal in the open-ended questions.
- Teachers-pre: deletion of item 2, due to its CVR, in addition to the fact that the means in representativeness and relevance pointed to a need for reformulation.
- Teachers-post: reformulation of item 2 due to its representativeness, relevance and formulation means. Former item 2 has been split into two new items.

Despite the modifications, all the questionnaires in version (v2) measure the dimensions proposed in Table 2, except the initial questionnaire for students, which includes a new dimension, the perception of competence (self-efficacy). In addition, there are some changes in the number of items, as the initial questionnaire for students goes from 3 to 4 items, the initial questionnaire for teachers reduces one item in (from 6 to 5) and the final questionnaire for teachers increases in one item (from 16 to 17). The design and feature of the questionnaires in version (v2) has been given in Table 9.

Once the objective of designing the instruments in phase I has been achieved and sufficient evidence of content validity has been obtained in this expert judgment, the analysis of the questionnaires in version (v2) has been carried out in a pilot sample. The pilot sample contains students from all pre-university academic cycles (primary, secondary), is gender balanced in line with the inclusive spirit of the project, and the schools are located in diverse contexts (from small urban centers to large cities).

The results regarding the evidence of reliability in the applied sample suggest that there is sufficient internal consistency of the Likert-type items included in each of the questionnaires. After the qualitative analysis of the remaining items, it is concluded that they have been answered in their entirety, in accordance with the purpose for which they were designed, so that the administration of the questionnaires to the pilot sample allows us to conclude that the objective of phase II has been achieved. In spite of this, it is necessary to modify some of the response formats. Specifically, in the initial student questionnaire, item 4 has changed from an open-ended question to a multiple-choice response to avoid the broad range of responses that has been observed when processing the qualitative analysis. The same happens with item 2 of the final questionnaire for students and teachers. In addition, item 6 of the teachers-post questionnaire adds the suggestion “justify your answer” to improve the quality of the gathered data. As a result of phase II, the version (v3) of the five questionnaires has been obtained, where the students-pre questionnaire, and the teachers-pre and teachers-post questionnaires have been modified as discussed above with respect to version (v2).

The set of questionnaires, in their final version (v3), are a valuable resource for the evaluation of the *family* action of the Girls4STEM initiative, allowing to assess the impact over all target audiences (students, families and teachers). The mixed methods methodology has allowed to refine the set of instruments through the use of different techniques, such as the expert judgment. Moreover, the analysis of the set of instruments administered to a pilot sample of the study population has enabled the collection of evidence that survey items and response options are well understood by respondents.

This set of instruments has been designed and validated with the aim of overcoming the challenges faced by the evaluation of informal STEM education actions. On the one hand, the instruments incorporate features in the evaluation that are often overlooked, such as improvement of the initiative, with measures at different times, e.g., pre and post action for students and teachers. On the other hand, completing the questionnaires does not require excessive time due to their well-designed formulation, which maximizes the likelihood that they will be completed properly by the participants, including primary students from lower courses which might be less familiar with filling on-line forms without help. The fact that they can be delivered on-line, simplifies the posterior data analysis and contributes to the sustainability of the initiative. In addition, preliminary reliability and validity evidence conducted by a multidisciplinary team of researchers has been provided, which to the best of our knowledge, positions this work as a core reference in informal STEM education contexts. Although the initiative Girls4STEM is located in Spain, the process followed to achieved the set of instruments in version (v3) can be applied to any informal evaluation initiative with a low-cost implementation. Moreover, the set of instruments is openly offered for review or administration in other educational experiences in informal education, so that particular features of different cultural contexts can be incorporated via each initiative's objectives. Nevertheless, it is desirable to continue researching and collecting new evidence in on-going and future editions of the initiative, in order to continue improving the rigor of the questionnaires, being applied to other samples or adapted for administration to other STEM educational projects.

## Data Availability Statement

The original contributions presented in the study are included in the article/Supplementary Material, further inquiries can be directed to the corresponding authors.

## Ethics Statement

Ethical review and approval was not required for the study on human participants in accordance with the local legislation and institutional requirements. Written informed consent from the patients/ participants or patients/participants legal guardian/next of kin was not required to participate in this study in accordance with the national legislation and the institutional requirements.

## Author Contributions

MH-P designed the research. CB-M, EV, and XB collected the data. MH-P, CB-M, EV, EL-I, AF, XB, and SR analyzed the research. MH-P, CB-M, and EL-I searched the literature. MH-P, CB-M, EV, and EL-I wrote the manuscript. All authors contributed to the article and approved the submitted version.

## Funding

This research was partially supported by the project FCT-20-15904 from the Fundación Española para la Ciencia y la Tecnología (FECYT) and the Ministerio de Ciencia e Innovación and the project GV/2021/110 from Generalitat Valenciana.

## Conflict of Interest

The authors declare that the research was conducted in the absence of any commercial or financial relationships that could be construed as a potential conflict of interest.

## Publisher's Note

All claims expressed in this article are solely those of the authors and do not necessarily represent those of their affiliated organizations, or those of the publisher, the editors and the reviewers. Any product that may be evaluated in this article, or claim that may be made by its manufacturer, is not guaranteed or endorsed by the publisher.

## Footnotes

^1^Ministerio de Universidades. Students statistics. https://bit.ly/3yA6Bcs.
