# Supplementary material for: On the Design and Validation of Assessing Tools for Measuring the Impact of Programs Promoting STEM Vocations
Source: Front Psychol. 2022 Jun 27;13:937058. doi: 10.3389/fpsyg.2022.937058 (PMC9291434; doi:10.3389/fpsyg.2022.937058)
Supplement: Supplementary file 1 [file Data_Sheet_1.pdf]

frontiers

Supplementary Material

In this supplementary material, we provide the following information:

- Questionnaires (v1): parents, students pre, students post, teachers pre, teachers post
- Questionnaires (v2): parents, students pre, students post, teachers pre, teachers post
- Questionnaires (v3): students pre, students post, teachers post

Note that all the questionnaires included the acceptance of the use of personal data according to the Spanish regulation[1] as well as a short description of the project aims and main funding agencies, which was adapted depending on the target audience (students, teachers or families).

[1] https://www.uv.es/girls4stem/politica-de-privacidad/FichaResumenRegistroActividad.pdf

1 Questionnaires (v1)

Parents

<u>Dimension: Overall impact</u>

1) What do you feel the activity has contributed to the student? (Select all that apply)

	Getting to know STEM professions at first hand
	Increase their interest in STEM professions
	Become more aware of gender stereotypes related to STEM professions
	Nothing
	Other

2) What has the activity brought to yourself? (Select all that apply)

	Getting to know STEM professions at first hand
	Increase their interest in STEM professions
	Become more aware of gender stereotypes related to STEM professions
	Other

3) Does your family encourage your son's or daughter's participation in activities related to science, technology, engineering and/or mathematics?

Yes	
No	

If yes, select all that apply: visiting museums, visiting science centers, STEM workshops (programming, robotics, mathematics, experiments...), STEM extracurriculars (programming, robotics, mathematics, experiments...), attending STEM talks or conferences, providing STEM resources at home (microscope, robots, logic games...), reading (books, magazines, comics...) about science, technology, engineering and/or mathematics and other.

Dimension: Impact on parents

4) I now feel more interest about new topics in science, technology, engineering, or mathematics.

	1	2	3	4	5	
Not at all in agreement						Totally in agreement

5) The activity has made me reflect on the role of women in these professions.

	1	2	3	4	5	
Not at all in agreement						Totally in agreement

6) After the activity, I now consider it more important to encourage interest in STEM activities to the student and/or the student I am accompanying.

	1	2	3	4	5	
Not at all in agreement						Totally in agreement

7) After the activity, I consider it more important to bring female STEM figures closer to the student I am accompanying.

	1	2	3	4	5	
Not at all in agreement						Totally in agreement

Dimension: Satisfaction and project improvement

8) I found the activity satisfactory in general.

	1	2	3	4	5	
Not at all in agreement						Totally in agreement

9) What aspects of the activity do you value most positively?

10) What do you think could be improved about this activity?

Students pre

<u>Dimension: STEM interests</u>

1) Think about the activities you like to do in your free time. Indicate if any of them are related to science, technology, engineering, or mathematics.

Yes	
No	

If yes, state what these activities are (e.g., field trips to science centers, museums, programming, reading about science and experiments, etc.).

2) Indicate how interested you are in each of these subjects, where 1 indicates "not at all interested" and 5 indicates "very interested".

<u>Mathematics</u>

	1	2	3	4	5	
I am not interested at all						I am very much interested

<u>Natural sciences</u>

	1	2	3	4	5	
I am not interested at all						I am very much interested

<u>Dimension: Achievement in STEM subjects</u>

3) Indicate your grade (mark) in the last evaluation of the following subjects.

<u>Mathematics</u>

<u>Natural sciences</u>

Students post

<u>Dimension: Degree of participation</u>

1) Indicate the reason(s) why you have decided to participate in this activity.
2) What aspects of this activity do you consider most important and why?

<u>Dimension: Impact on students</u>

3) I am now more interested in science, technology, engineering, or mathematics.

	1	2	3	4	5	
Not at all in agreement						Totally in agreement

4) The activity has allowed me to learn new information about these professions.

	1	2	3	4	5	
Not at all in agreement						Totally in agreement

5) I am more interested than before in studying about STEM fields in the future.

	1	2	3	4	5	
Not at all in agreement						Totally in agreement

6) After the activity, I believe that women could be more involved in STEM professions.

	1	2	3	4	5	
Not at all in agreement						Totally in agreement

<u>Dimension: Satisfaction and project improvement</u>

7) I found the activity satisfactory overall

	1	2	3	4	5	
Not at all in agreement						Totally in agreement

8) What aspects of the project do you value most positively? In other words, what did you like most about the project?

9) What do you think could be improved?

Teachers pre

Dimension: Motivation towards the project

1) Indicate the reason(s) why you have decided to participate in this project.
2) What aspects do you find most important and why?

Dimension: Expectations (students)

3) What do you think participation in the project will bring to your students?
4) What image of STEM disciplines would you want your students to have after participating in the project?
5) How do you think your students' participation in the project will impact them regarding STEM (e.g., knowledge, interests, and views about women in STEM professions)?

Dimension: Expectations (teachers)

6) How do you think the participation in this project (as a teacher) will affect your professional practice?

Teachers post

Dimension: Degree of participation

1) Explain how you have been involved in the project.
2) How much time have you spent on it and how much time have your students spent on it?

Dimension: Impact on students

3) What do you think participation in the project has brought to your students?
4) What image of STEM disciplines do you think your students now have after participating in the project?
5) How do you think your students' participation has affected them regarding STEM (e.g., knowledge, interests, and views about women in STEM professions)?

Dimension: Impact on teachers

6) What has participation in this project contributed to your professional practice as a teacher?
7) After participating in the project, do you consider yourself to be able to carry out similar activities with your students?

Yes	
Yes, but with some limitations	
No	

8) I am now more interested in new topics in science, technology, engineering, or mathematics.

	1	2	3	4	5	
Not at all in agreement						Totally in agreement

9) The activity has made me reflect on the presence of women in these professions.

	1	2	3	4	5	
Not at all in agreement						Totally in agreement

10) After the activity, I now consider it more important to encourage interest in STEM activities in my students.

	1	2	3	4	5	
Not at all in agreement						Totally in agreement

11) After the activity I consider it more relevant to include female STEM referents in my didactic programs.

	1	2	3	4	5	
Not at all in agreement						Totally in agreement

12) I will modify my didactic programs to incorporate female STEM referents in my subject.

	1	2	3	4	5	
Not at all in agreement						Totally in agreement

13) I feel that I have enough training to make changes in my didactic programs that favor the inclusion of female STEM referents in the content.

	1	2	3	4	5	
Not at all in agreement						Totally in agreement

Dimension: Satisfaction and project improvement

14) I found the activity satisfactory overall

	1	2	3	4	5	
Not at all in agreement						Totally in agreement

15) What aspects of the project do you value most positively?

16) What do you think could be improved in this project?

2 Questionnaires (v2).

Parents

Dimension: Overall impact

1) What do you feel the activity has contributed to the student? (Select all that apply)

	Getting to know STEM professions at first hand
	Increase their interest in STEM professions
	Become more aware of gender stereotypes related to STEM professions
	Nothing
	Other

2) What has the activity brought to your family? (Select all that apply)

	Getting to know STEM professions at first hand
	Increase their interest in STEM professions
	Become more aware of gender stereotypes related to STEM professions
	Other

3) Does your family encourage your son's or daughter's participation in activities related to science, technology, engineering and/or mathematics?

Yes	
No	

If yes, select all that apply: visiting museums, visiting science centers, STEM workshops (programming, robotics, mathematics, experiments...), STEM extracurriculars (programming, robotics, mathematics, experiments...), attending STEM talks or conferences, providing STEM resources at home (microscope, robots, logic games...), reading (books, magazines, comics...) about science, technology, engineering and/or mathematics and other.

<u>Dimension: Impact on parents</u>

4) I now feel more interest about new topics in science, technology, engineering, or mathematics.

	1	2	3	4	5	
Not at all in agreement						Totally in agreement

5) The activity has made me reflect on the participation of women in these professions.

	1	2	3	4	5	
Not at all in agreement						Totally in agreement

6) After the activity, I now consider it more important to encourage interest in STEM activities to the student and/or the student I am accompanying.

	1	2	3	4	5	
Not at all in agreement						Totally in agreement

7) After the activity, I consider it more important to bring female STEM figures closer to the student I am accompanying.

	1	2	3	4	5	
Not at all in agreement						Totally in agreement

<u>Dimension: Satisfaction and project improvement</u>

8) I found the activity satisfactory in general.

	1	2	3	4	5	
Not at all in agreement						Totally in agreement

We work every day to keep improving. Your opinion helps us to achieve it.

9) What aspects of the activity do you value most positively?

10) What do you think could be improved about this activity?

Students pre

Dimension: STEM interests

1) Think about the activities you like to do in your free time. Indicate if any of them are related to science, technology, engineering, or mathematics.

Yes	
No	

If yes, state what these activities are (e.g., field trips to science centers, museums, programming, reading about science and experiments, etc.).

2) Indicate how interested you are in each of these subjects, where 1 indicates "not at all interested" and 5 indicates "very interested".

Version for primary students (6-12 years old)

Mathematics

	1	2	3	4	5	
I am not interested at all						I am very much interested

Natural sciences

	1	2	3	4	5	
I am not interested at all						I am very much interested

Version for secondary students (12-18 years old)

Mathematics

	1	2	3	4	5	
I am not interested at all						I am very much interested

Biology and Geology

	1	2	3	4	5	
I am not interested at all						I am very much interested

Physics and Chemistry

	1	2	3	4	5	
I am not interested at all						I am very much interested

Dimension: self-efficacy (perceived achievement)

3) Indicate how good you are at each of the above subjects, where 1 indicates "not good at all" and 5 indicates "very good".

Version for primary students (6-12 years old)

Mathematics

	1	2	3	4	5	
Not good at all						Very good

Natural sciences

	1	2	3	4	5	
Not good at all						Very good

Version for secondary students (12-18 years old)

Mathematics

	1	2	3	4	5	
Not good at all						Very good

Biology and Geology

	1	2	3	4	5	
Not good at all						Very good

Physics and Chemistry

	1	2	3	4	5	
Not good at all						Very good

Dimension: Achievement in STEM subjects

4) Indicate your grade (mark) in the last evaluation of the following subjects.

Version for primary students (6-12 years old)

Mathematics

Natural sciences

Version for secondary students (12-18 years old)

Mathematics

Biology and Geology

Physics and Chemistry

Students post

Dimension: Degree of participation

1) Explain what your participation in the project consisted of. What did you do?
2) How much time have you spent on it (total number of hours)?

Now that you have participated in the project, we would like you to answer a series of questions rated from 1 to 5, where 1 indicates “not at all in agreement” and 5 “totally in agreement”

Dimension: Impact on students

1) I am now more interested in science, technology, engineering, or mathematics.

	1	2	3	4	5	
Not at all in agreement						Totally in agreement

2) I am more interested than before in studying about STEM fields in the future.

	1	2	3	4	5	
Not at all in agreement						Totally in agreement

3) The activity has allowed me to learn new information about these professions.

	1	2	3	4	5	
Not at all in agreement						Totally in agreement

4) After the activity, I believe that women could be more involved in STEM professions.

	1	2	3	4	5	
Not at all in agreement						Totally in agreement

Dimension: Satisfaction and project improvement

5) I found the activity satisfactory overall

	1	2	3	4	5	
Not at all in agreement						Totally in agreement

6) What aspects of the project do you value most positively? In other words, what did you like most about the project?
7) What do you think could be improved?

Teachers pre

Dimension: Motivation towards the project

3) Indicate the reason(s) why you have decided to participate in this project.

Dimension: Expectations (students)

4) What do you think participation in the project will bring to your students?
5) What image of STEM disciplines would you want your students to have after participating in the project?
6) How do you think your students' participation in the project will impact them regarding STEM (e.g., knowledge, interests, and views about women in STEM professions)?

Dimension: Expectations (teachers)

7) How do you think the participation in this project (as a teacher) will affect your professional practice?

Teachers post

Dimension: Degree of participation

1) Explain how you have been involved in the project.
2) Indicate the number of hours you have spent.
3) Indicate the number of videos you have participated in.

0	1	2	3	4

Dimension: Impact on students

4) What do you think participation in the project has brought to your students?
5) What image of STEM disciplines do you think your students now have after participating in the project?
6) How do you think your students' participation has affected them regarding STEM (e.g., knowledge, interests, and views about women in STEM professions)?

Dimension: Impact on teachers

7) What has participation in this project contributed to your professional practice as a teacher?
8) After participating in the project, do you consider yourself to be able to carry out similar activities with your students?

Yes	
Yes, but with some limitations	
No	

9) I am now more interested in new topics in science, technology, engineering, or mathematics.

	1	2	3	4	5	
Not at all in agreement						Totally in agreement

10) The activity has made me reflect on the presence of women in these professions.

	1	2	3	4	5	
Not at all in agreement						Totally in agreement

11) After the activity, I now consider it more important to encourage interest in STEM activities in my students.

	1	2	3	4	5	
Not at all in agreement						Totally in agreement

12) After the activity I consider it more relevant to include female STEM referents in my didactic programs.

	1	2	3	4	5	
Not at all in agreement						Totally in agreement

13) I will modify my didactic programs to incorporate female STEM referents in my subject.

	1	2	3	4	5	
Not at all in agreement						Totally in agreement

14) I feel that I have enough training to make changes in my didactic programs that favor the inclusion of female STEM referents in the content.

	1	2	3	4	5	
Not at all in agreement						Totally in agreement

Dimension: Satisfaction and project improvement

15) I found the activity satisfactory overall

	1	2	3	4	5	
Not at all in agreement						Totally in agreement

16) What aspects of the project do you value most positively?

17) What do you think could be improved in this project?

3 Questionnaires (v3).

Students pre

Dimension: STEM interests

1) Think about the activities you like to do in your free time. Indicate if any of them are related to science, technology, engineering, or mathematics.

Yes	
No	

If yes, state what these activities are (e.g., field trips to science centers, museums, programming, reading about science and experiments, etc.).

2) Indicate how interested you are in each of these subjects, where 1 indicates "not at all interested" and 5 indicates "very interested".

Version for primary students (6-12 years old)

Mathematics

	1	2	3	4	5	
I am not interested at all						I am very much interested

Natural sciences

	1	2	3	4	5	
I am not interested at all						I am very much interested

Version for secondary students (12-18 years old)

Mathematics

	1	2	3	4	5	
I am not interested at all						I am very much interested

Biology and Geology

	1	2	3	4	5	
I am not interested at all						I am very much interested

Physics and Chemistry

	1	2	3	4	5	
I am not interested at all						I am very much interested

Dimension: self-efficacy (perceived achievement)

3) Indicate how good you are at each of the above subjects, where 1 indicates "not good at all" and 5 indicates "very good".

Version for primary students (6-12 years old)

Mathematics

	1	2	3	4	5	
Not good at all						Very good

Natural sciences

	1	2	3	4	5	
Not good at all						Very good

Version for secondary students (12-18 years old)

Mathematics

	1	2	3	4	5	
Not good at all						Very good

Biology and Geology

	1	2	3	4	5	
Not good at all						Very good

Physics and Chemistry

	1	2	3	4	5	
Not good at all						Very good

Dimension: Achievement in STEM subjects

4) Indicate your grade (mark) in the last evaluation of the following subjects.

Version for primary students (6-12 years old)

Mathematics

- Between 0 and 3
- Between 3.1 and 4.9
- Between 5 and 5.9
- Between 6 and 6.9
- Between 7 and 8.9

- Between 9 and 10

Natural sciences

- Between 0 and 3
- Between 3.1 and 4.9
- Between 5 and 5.9
- Between 6 and 6.9
- Between 7 and 8.9
- Between 9 and 10

Version for secondary students (12-18 years old)

Mathematics

- Between 0 and 3
- Between 3.1 and 4.9
- Between 5 and 5.9
- Between 6 and 6.9
- Between 7 and 8.9
- Between 9 and 10
- I do not take this course

Biology and Geology

- Between 0 and 3
- Between 3.1 and 4.9
- Between 5 and 5.9
- Between 6 and 6.9
- Between 7 and 8.9
- Between 9 and 10
- I do not take this course

Physics and Chemistry

- Between 0 and 3
- Between 3.1 and 4.9
- Between 5 and 5.9
- Between 6 and 6.9
- Between 7 and 8.9
- Between 9 and 10
- I do not take this course

Students post

Dimension: Degree of participation

1) Explain what your participation in the project consisted of. What did you do?
2) How much time have you spent on it (total number of hours)?
   - Between 0 and 1 hour
   - Between 1 and 2 hours
   - Between 2 and 3 hours
   - Between 3 and 4 hours
   - Between 4 and 5 hours
   - Between 5 and 6 hours
   - Between 6 and 7 hours
   - Between 7 and 8 hours
   - Between 8 and 10 hours
   - Between 10 and 15 hours
   - More than 15 hours

Now that you have participated in the project, we would like you to answer a series of questions rated from 1 to 5, where 1 indicates "not at all in agreement" and 5 "totally in agreement"

<u>Dimension: Impact on students</u>

3) I am now more interested in science, technology, engineering, or mathematics.

	1	2	3	4	5	
Not at all in agreement						Totally in agreement

4) I am more interested than before in studying about STEM fields in the future.

	1	2	3	4	5	
Not at all in agreement						Totally in agreement

5) The activity has allowed me to learn new information about these professions.

	1	2	3	4	5	
Not at all in agreement						Totally in agreement

6) After the activity, I believe that women could be more involved in STEM professions.

	1	2	3	4	5	
Not at all in agreement						Totally in agreement

<u>Dimension: Satisfaction and project improvement</u>

7) I found the activity satisfactory overall

	1	2	3	4	5	
Not at all in agreement						Totally in agreement

8) What aspects of the project do you value most positively? In other words, what did you like most about the project?
9) What do you think could be improved?

Teachers post

Dimension: Degree of participation

1) Explain how you have been involved in the project.

2) Indicate the number of hours you have spent.
   - Between 0 and 1 hour
   - Between 1 and 2 hours
   - Between 2 and 3 hours
   - Between 3 and 4 hours
   - Between 4 and 5 hours
   - Between 5 and 6 hours
   - Between 6 and 7 hours
   - Between 7 and 8 hours
   - Between 8 and 10 hours
   - Between 10 and 15 hours
   - More than 15 hours

3) Indicate the number of videos you have participated in.

0	1	2	3	4

<u>Dimension: Impact on students</u>

4) What do you think participation in the project has brought to your students?
5) What image of STEM disciplines do you think your students now have after participating in the project?
6) How do you think your students' participation has affected them regarding STEM (e.g., knowledge, interests, and views about women in STEM professions)? Justify your answer.

<u>Dimension: Impact on teachers</u>

7) What has participation in this project contributed to your professional practice as a teacher?
8) After participating in the project, do you consider yourself to be able to carry out similar activities with your students?

Yes	
Yes, but with some limitations	
No	

9) I am now more interested in new topics in science, technology, engineering, or mathematics.

	1	2	3	4	5	
Not at all in agreement						Totally in agreement

10) The activity has made me reflect on the presence of women in these professions.

	1	2	3	4	5	
Not at all in agreement						Totally in agreement

11) After the activity, I now consider it more important to encourage interest in STEM activities in my students.

	1	2	3	4	5	
Not at all in agreement						Totally in agreement

12) After the activity I consider it more relevant to include female STEM referents in my didactic programs.

	1	2	3	4	5	
Not at all in agreement						Totally in agreement

13) I will modify my didactic programs to incorporate female STEM referents in my subject.

	1	2	3	4	5	
Not at all in agreement						Totally in agreement

14) I feel that I have enough training to make changes in my didactic programs that favor the inclusion of female STEM referents in the content.

	1	2	3	4	5	
Not at all in agreement						Totally in agreement

Dimension: Satisfaction and project improvement

15) I found the activity satisfactory overall

	1	2	3	4	5	
Not at all in agreement						Totally in agreement

16) What aspects of the project do you value most positively?

17) What do you think could be improved in this project?
